# Genetic insights into Shewanella spp., progenitor of the blaOXA-48-like genes: a large-scale study

**DOI:** 10.1099/mgen.0.001417

**Published:** 2025-06-05

**Authors:** Ning Dong, Yanyan Zhang, Yuchen Wu, Xiaoyang Ju, Zelin Yan, Congcong Liu, Jiaxing Yang, Hongwei Zhou, Gongxiang Chen, Sheng Chen, Rong Zhang

**Affiliations:** 1 Department of Clinical Laboratory, Second Affiliated Hospital of Zhejiang University, School of Medicine, Hangzhou, Zhejiang, PR China; 2 School of Public Health, Zhejiang University School of Medicine, Hangzhou, Zhejiang, PR China; 3 State Key Lab for Chemical Biology and Drug Discovery, and the Department of Food Science and Nutrition, The Hong Kong Polytechnic University, Hung Hom, Kowloon, Hong Kong, SAR

**Keywords:** genomic characterization, OXA-48-like, progenitor, *Shewanella *spp., transmission

## Abstract

*Shewanella* spp. played pivotal ecological roles and were reported to be the progenitor of *bla*_OXA-48_-like carbapenemase genes. However, it remained unknown which species was the progenitor of different OXA-48 carbapenemase variants. To address this issue, we analysed the largest collection of *Shewanella* genomes to our knowledge and performed genetic and phenotypic analysis on *Shewanella* collected from Zhejiang province, China. Our results suggested that *bla*_OXA-48_-like was intrinsically carried by a few *Shewanella* species and different *bla*_OXA-48_-like variants were associated with different *Shewanella* species; for instance, *Shewanella baltica* was associated with *bla*_OXA-924_, and some *Shewanella oncorhynchi* and *Shewanella putrefaciens* carried *bla*_OXA-900_-like. The *bla*_OXA-48_-like genes carried by *Shewanella xiamenensis* were highly diverse. Comparatively, none of the *Shewanella algae* genomes carried *bla*_OXA-48_-like. Results of phylogenetic analysis supported the notion that OXA-48-like carbapenemase originated from different environmental *Shewanella* species and was captured by clinical species, particularly *Enterobacterales*. Different *Shewanella* species distributed in different niches in Zhejiang province, i.e. *S. algae* (*n*=12) and *Shewanella indica* (*n*=1) strains were all isolated from clinical settings and *S. xiamenensis* (*n*=23) and *Shewanella mangrovisoli* (*n*=2) were isolated from both hospital sewage and river water. *bla*_OXA-48-like_ genes in *Shewanella* spp. from Zhejiang province were located on the chromosome. To the best of our knowledge, this is the first study investigating the progenitor of different *bla*_OXA-48_-like variants with a focus on the *Shewanella* population. Results in this study highlighted the important role of *Shewanella* species in the ecosystem, particularly as the major source of the notorious carbapenemase gene, *bla*_OXA-48_. Control measures should be implemented to prevent further dissemination of such organisms in the hospital setting and the community.

**Impact Statement**

*Shewanella* sp. is an important environmental species playing pivotal ecological roles, with several members being pathogenic to human beings. Through genomic analysis of all available *Shewanella* genomes and epidemiological study of *Shewanella* strains from Zhejiang province, China, a few important conclusions were achieved, including the following: (1) *bla*_OXA-48_-like gene was intrinsically carried by some *Shewanella* species; (2) *bla*_OXA-48_-like originated from environmental *Shewanella* and was captured by *Enterobacterales*; (3) the distribution of *Shewanella* was niche specific in Zhejiang province, either environmentally or clinically; and (4) *bla*_OXA-48-like_ in *Shewanella* was chromosome-borne. Results in this study highlighted the important role of *Shewanella* species in the ecosystem, particularly as the major source of the notorious carbapenemase gene, *bla*_OXA-48_.

## Data Summary

The whole-genome sequencing data of all strains in this study have been submitted to the National Center for Biotechnology Information (NCBI) database under the BioProject accession number PRJNA1064064.

## Introduction

*Shewanella* is a unique member of the *Shewanellaceae* family, which includes facultatively anaerobic Gram-negative rods [1]. As of March 2024, a total of 130 species have been identified in this genus (https://lpsn.dsmz.de/search?word=Shewanella). *Shewanella* is commonly considered an environmental genus widely distributed in nature, particularly in aquatic environments including seawater, freshwater and in marine organisms [2]. Some members of this genus, such as *Shewanella oneidensis*, have been identified that could potentially play beneficial roles in ecological processes such as serving as microbial fuel cells and bioremediation of toxic agents [3]. In addition, some species like *Shewanella putrefaciens*, *Shewanella algae*, *Shewanella haliotis* and *Shewanella xiamenensis* have been documented with pathogenicity in human beings [4]. Reported illnesses related to *Shewanella* include bacteremia, pneumonia, endocarditis, otitis media and soft tissue, skin, intracerebral, ocular and gastrointestinal infections, some of which could lead to serious clinical or even fatal consequences [5,7].

Antibiotic resistance is reported to be a natural phenomenon that predates the modern selective pressure of clinical antibiotic use [8]. The origin of many modern resistance genes in pathogens is likely environmental bacteria [9]; for instance, *Acinetobacter radioresistens* was the progenitor of the *bla*_OXA-23_ CHDL gene widespread in *A. baumannii* [10], *Flavobacteriaceae* was the potential ancestral source of tetracycline resistance gene *tet*(X) [11] and the RND efflux gene cluster *tmexCD-toprJ* conferring multidrug resistance originated from the chromosome of *Pseudomonas* [12]. Likewise, bacteria of the environmental genus, *Shewanella*, are a reservoir of antibiotic resistance determinants. Particularly, *Shewanella* was the progenitor of the Qnr-type quinolone resistance genes and the *bla*_OXA-48-like_ oxacillinase class D β-lactamase-encoding genes conferring carbapenem resistance [2].

OXA-48-like carbapenemases are the most common carbapenemases in the clinically important pathogens, *Enterobacterales*, in certain regions of the world (e.g. the Middle East, North Africa and some European countries) [13]. The OXA-48-like carbapenemases are highly diverse, among which OXA-48, OXA-181, OXA-232, OXA-204, OXA-162 and OXA-244 are the most commonly reported [13]. *Shewanella* spp. were considered important reservoirs and progenitors of carbapenem-hydrolysing OXA enzymes, including OXA-48-like carbapenemase, which was the most common carbapenemase in *Enterobacterales* in certain regions of the world [13]. The first chromosome-encoded OXA carbapenemase in *Shewanella* spp., OXA-54, whose potential source could be *S. oneidensis*, was reported around the same time as OXA-48 [14]. Different *Shewanella* species could carry different *bla*_OXA-48_-like variants, for example, several variants including *bla*_OXA-48_, *bla*_OXA-199_, *bla*_OXA-204_ and *bla*_OXA-181_ were identified in *S. xiamenensis*, and *bla*_OXA-54_ was identified in *S. oneidensis* [14,18]. Mobile genetic elements, such as the composite transposon, Tn*1999,* were associated with the mobilization of OXA-48-like *β*-lactamase from the chromosome of *Shewanella* spp. to other bacteria, including *Enterobacterales*, posing a serious threat to human health [19]. Nevertheless, the genetic characteristics of the *Shewanella* population, the distribution and the transmission potential of *bla*_OXA-48_-like carbapenemase genes in *Shewanella* remained poorly understood. In this study, we conducted a comprehensive genomic characterization with genome sequences from both the National Center for BiotechnologyInformation (NCBI) database and our collection to fill this knowledge gap.

## Methods

### Genomic sequence retrieval, species identification and genetic characterization

All *Shewanella* spp. genomes publicly available in the NCBI Genome database (https://www.ncbi.nlm.nih.gov/genome/genomes/) as of 1 September 2023 were downloaded. FastANI was used to calculate the whole-genome average nucleotide identity (ANI) between the downloaded genomes and the reference sequences of the 130 *Shewanella* species available in the List of Prokaryotic names with Standing in Nomenclature database [20]. Species were identified based on the ANI values. The presence of *bla*_OXA-48_-like genes and other acquired antimicrobial resistance genes was identified using ResFinder 4.1 [21].

### Phylogenetic analysis of *Shewanella* spp*.* from the database

All *Shewanella* spp. genomes from the NCBI database were screened for the presence of 40 conserved single-copy genes using FetchMG v1.0 [22]. These markers were translated into protein sequences, concatenated and aligned using muscle v5.1. The aligned amino acid sequence was trimmed using trimal v1.2rev59 [23]. FastTree v2.1.11 was used to construct an approximately maximum likelihood tree with default parameters [24]. The generated phylogenetic tree was visualized and edited with iTOL version 6 [25].

### Sequence acquisition and alignment of the OXA-48-like protein

The amino acid sequence of all OXA-48-like carbapenemases available as of 1 December 2023 was downloaded from both the Comprehensive Antibiotic Resistance Database (https://card.mcmaster.ca/) and the Pathogen Detection Reference Gene Catalog database. Multiple sequence alignment was performed using muscle v3.8.31 with default parameters [26]. A model with the least score (JTT+G) evaluated using the Models function in mega 7 was selected as the best amino acid substitution model to reconstruct the maximum likelihood tree [27]. Gaps and missing data were treated as partial deletions. Results were validated using 1,000 bootstrap replicates [27].

### Strain collection and species identification

*Shewanella* strains were collected during the period 2022–2023 as part of an ongoing surveillance programme aimed at identifying Gram-negative organisms in Zhejiang province, China. Samples were collected from different sources in Zhejiang, including clinical settings, rivers and hospital sewages. Clinical samples, including pustule, sputum and blood, were collected from patients hospitalized in The Second Affiliated Hospital of Zhejiang University School of Medicine (Jiefang Road). The hospital is one of the major medical centres in Zhejiang province and a large comprehensive tertiary hospital. In 2022, the annual outpatient volume of the hospital exceeded 5 million visits, reflecting its role as a leading medical centre in the region. Hospital sewage samples were collected from six hospitals in Zhejiang. River water samples were collected from four rivers in Zhejiang province. The geographic distributions of the sampling sites were listed in Table S4 (available in online Supplementary Material). Strains were isolated on LB agar plates containing 0.3 µg ml^−1^ meropenem. All strains were subjected to MALDI-TOF MS (Bruker Daltonik GmbH, Bremen, Germany). Subsequently, strains identified as belonging to *Shewanella* spp. were subjected to WGS to determine the exact species and facilitate further research.

### Antimicrobial susceptibility testing

The minimum inhibitory concentrations (MICs) of all *Shewanella* strains against 15 commonly used antibiotics (imipenem, meropenem, ertapenem, cefmetazole, ceftazidime, cefotaxime, piperacillin/tazobactam, cefoperazone/sulbactam, ceftazidime/avibactam, cefepime, polymyxin B, tigecycline, ciprofloxacin, amikacin and aztreonam) were determined by the VITEK 2 COMPACT automatic microbiology analyser and interpreted according to the Clinical and Laboratory Standards Institute guidelines, except tigecycline and colistin [28], the breakpoints of which were interpreted according to the European Committee on Antimicrobial Susceptibility Testing [29].

### Whole-genome sequencing and bioinformatics analysis

Genomic DNA was extracted from overnight cultures by using the PureLink Genomic DNA Mini Kit (Invitrogen, Carlsbad, CA, USA). All *Shewanella* strains were subjected to whole-genome sequencing using the HiSeq platform (Illumina, San Diego, CA). Genome assembly was conducted with SPAdes v3.15.5 [30]. Oxford nanopore MinION sequencing was conducted to obtain the complete genome sequences of selected strains carrying different OXA-48-like variants, using the SQK-NBD114.24 sequencing kit and flowcell R10.4.1. The raw sequencing data were basecalled using Guppy 3.0.3 with default parameters [31]. A hybrid assembly of Illumina and nanopore sequencing reads was constructed using Unicycler v 0.5.0 [32]. Genome sequences were annotated using RAST v2.0 with manual editing [33]. Acquired antibiotic resistance genes were identified by ResFinder 4.1 [34]. A heatmap of antimicrobial resistance genes was plotted using TBtools [35]. Insertion sequences (ISs) were identified using ISfinder v2.0 [36]. The genetic location (chromosome or plasmid) of the *bla*_OXA-48-like_ gene was determined by aligning the contigs carrying *bla*_OXA-48-like_ with complete genome sequences in the NCBI database and those generated in this study. The harvest suite was used to build a phylogenetic tree in this study with default parameters [37]. The core genomes of the strains were aligned, and phylogenetic trees were constructed using Parsnp v1.2, while SNPs in core genes among different strains were calculated using Gingr and snp-dists software. Briefly, Parsnp, which combines the advantages of both whole-genome alignment and read mapping, was used to produce a core-genome alignment, variant calls and a SNP tree. The tree construction function embedded in Parsnp was accomplished with FastTree2. The tree was visualized and edited with iTOL version 6 [25].

## Results

### *In silico* analysis of the genomic landscape of the *Shewanella* population

**Fig. 1.**
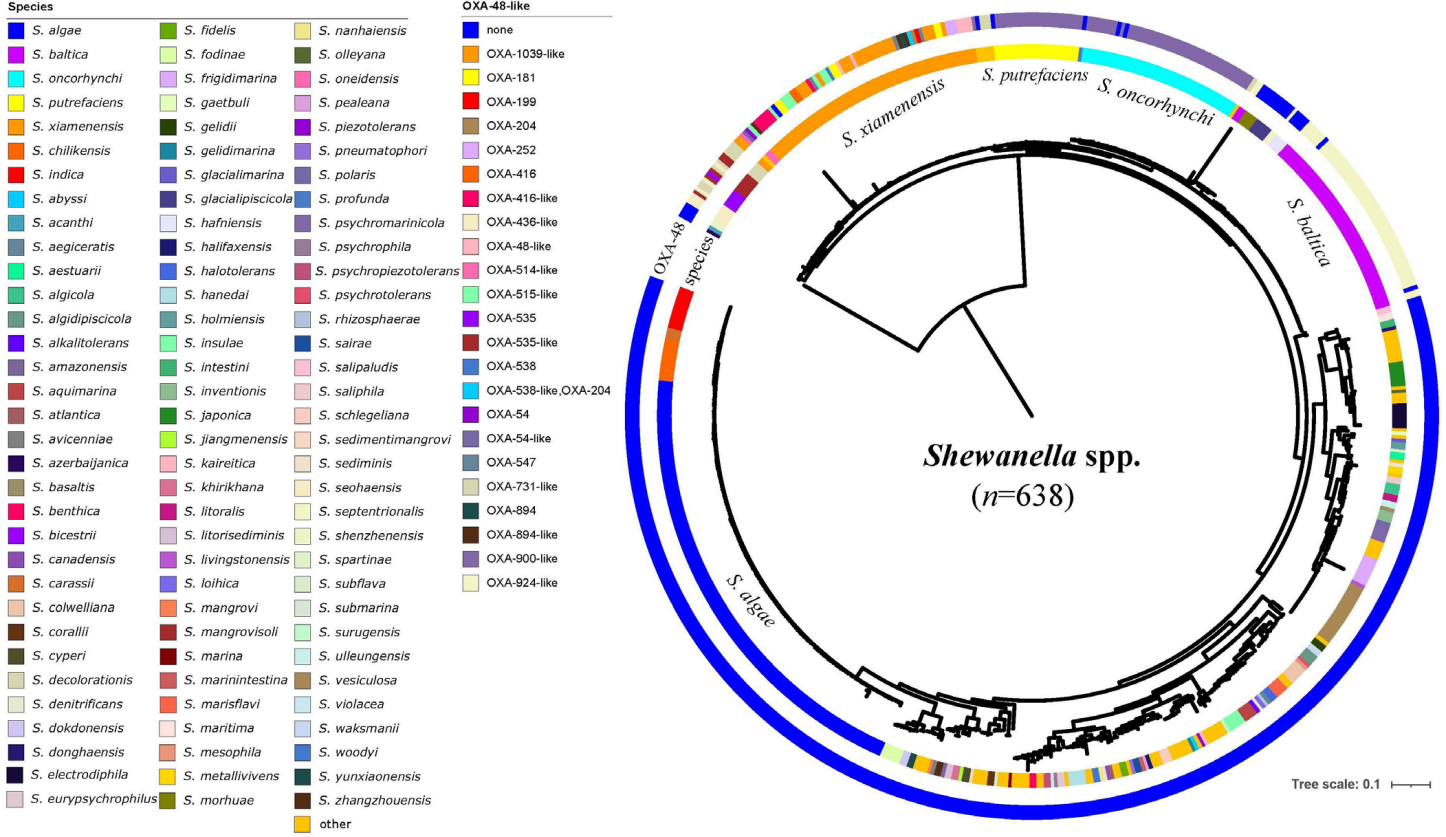
Phylogenetic tree of *Shewanella* spp. A total of 638 non-duplicated genome sequences were retrieved from the NCBI genome database and were used to build the phylogenetic tree. This tree was mid-rooted and visualized using iTOL. The circles from the innermost to outermost indicated the species and the production of OXA-48-like carbapenemase by each strain. The 72 strains that shared <95% identity with the reference genomes were labelled with ‘other’ to indicate that their species were not resolved.

*In silico* analyses were performed with a total of 638 non-duplicated *Shewanella* genomes retrieved from the NCBI database (query performed on 1 September 2023). The source for most of the 638 strains was unavailable and was thus not analysed. ANI analysis suggested that 72 out of the 638 genomes shared <95% ANI with that of their closest reference *Shewanella* species (ANI ranging from 77.56 to 94.82%), indicating they could belong to novel species not reported previously. The remaining 566 genomes covered 99 *Shewanella* species, among which 40 species were represented by only one genome. * S. algae* (*n*=130, 23.0%), *S. xiamenensis* (*n*=64, 11.3%), *Shewanella oncorhynchi* (*n*=46, 8.1%), *S. putrefaciens* (*n*=24, 4.2%) and *Shewanella vesiculosa* (*n*=18, 3.2%) accounted for the dominant species (Fig. 1, Table S1).

Among the 638 *Shewanella* genomes, a total of 216 (33.9%) carried *bla*_OXA-48_-like genes, including 18 different variants which were *bla*_OXA-900_-like (*n*=68), *bla*_OXA-924_-like (*n*=55), *bla*_OXA-1039_-like (*n*=24), *bla*_OXA-731_-like (*n*=12), *bla*_OXA-515_-like (*n*=8), *bla*_OXA-416_-like (*n*=8), *bla*_OXA-535_-like (*n*=6), *bla*_OXA-48_-like (*n*=6), *bla*_OXA-436_-like (*n*=6), *bla*_OXA-181_ (*n*=6), *bla*_OXA-54_-like (*n*=5), *bla*_OXA-894_-like (*n*=4), *bla*_OXA-252_ (*n*=3), *bla*_OXA-547_ (*n*=3), *bla*_OXA-538_-like (*n*=2), *bla*_OXA-204_ (*n*=2), *bla*_OXA-514_-like (*n*=1) and *bla*_OXA-199_ (*n*=1). Strains carrying *bla*_OXA-48_-like were genetically closely related compared with those without *bla*_OXA-48_-like genes (Fig. 1). The 216 genomes belonged to 11 different species, including *S. xiamenensis* (*n*=66), *Shewanella baltica* (*n*=55), *S. oncorhynchi* (*n*=46), *S. putrefaciens* (*n*=23), *Shewanella mangrovisoli* (*n*=6), *Shewanella seohaensis* (*n*=5), *Shewanella bicestrii* (*n*=5), *Shewanella decolorationis* (*n*=4), *S. oneidensis* (*n*=3), *Shewanella hafniensis* (*n*=2) and *Shewanella septentrionalis* (*n*=1). Among these, one *S. xiamenensis* carried two *bla*_OXA-48_-like genes, *bla*_OXA-538_-like and *bla*_OXA-204_.

Different variants of the *bla*_OXA-48_-like genes were associated with different *Shewanella* species, for instance, *bla*_OXA-924_-like genes were detected predominantly in *S. baltica* (54/55, 98.2%), and *bla*_OXA-900_-like genes were mostly detected in *S. oncorhynchi* (44/68, 64.7%) and *S. putrefaciens* (23/68, 33.8%). All except one *S. xiamenensis*, with a total number of 66, carried the *bla*_OXA-48_-like genes. The *bla*_OXA-48_-like genes carried by *S. xiamenensis* were highly diverse, including *bla*_OXA-1039_-like (*n*=24), *bla*_OXA-416_-like (*n*=8), *bla*_OXA-515_-like (*n*=8), *bla*_OXA-181_ (*n*=6), *bla*_OXA-48_-like (*n*=6), *bla*_OXA-894_-like (*n*=4), *bla*_OXA-252_ (*n*=3), *bla*_OXA-547_ (*n*=3), *bla*_OXA-538_-like (*n*=2), *bla*_OXA-204_ (*n*=2) and *bla*_OXA-199_ (*n*=1). Comparatively, none of the *S. algae* genomes carried * bla*_OXA-48_-like genes (Fig. 1, Table S1).

Other antimicrobial resistance genes carried by the 638 *Shewanella* genomes are shown in Table S6. A total of 24 strains (3.8%) carried *bla*_NDM-1_, which belonged to *S. xiamenensis* (*n*=10), *S. putrefaciens* (*n*=9), *S. bicestrii* (*n*=3) and *S. mangrovisoli* (*n*=2). Three *S. xiamenensis* carried *tet*(X4), among which two co-carried the RND efflux gene cluster *tmexCD3-toprJ3*. Besides, one *S. bicestrii* carried *bla*_VIM-2_. These findings suggested *Shewanella* could be an important vector of last-line antibiotic resistance genes. Other dominant resistance genes carried by these *Shewanella* strains were *bla*_OXA-SHE_ (*n*=134, 21.0%), *qnrA3* (*n*=53, 8.3%) and *qnrA7* (*n*=44, 6.9%), respectively.

### Origin, diversity and evolutionary trend of OXA-48-like carbapenemase

The G+C content of *Shewanella* spp. ranged from 40.23 to 55.69% (Table S2), with the median being 45.3% (https://www.ncbi.nlm.nih.gov/genome/13542). That of the *bla*_OXA-48_-like genes ranged from 44.11 to 47.37 %, with a median of 44.61% (Table S3). These data suggested the G+C content of *bla*_OXA-48_-like genes was similar to that of a few but not all *Shewanella* species, suggesting only a few *Shewanella* species could be the ancestral source of the * bla*_OXA-48_-like genes. This was in line with the finding that only 33.9% of *Shewanella* genomes carried *bla*_OXA-48_-like genes. According to the observation on the *bla*_OXA-48_ variants carried by different *Shewanella*, we speculated that *S. baltica* (G+C content: 46.31 mol%) could be the progenitor of *bla*_OXA-924_-like genes (45.36 mol%); *bla*_OXA-900_-like genes (44.53 mol%) could be originated from *S. oncorhynchi* (45.26 mol%) and/or *S. putrefaciens* (44.61 mol%); and *S. xiamenensis* (46.27 mol%) could be the ancestral source of several *bla*_OXA-48_ variants such as *bla*_OXA-1039_-like (44.49 mol%), *bla*_OXA-416_-like (44.61 mol%), *bla*_OXA-515_-like (44.74 mol%), *bla*_OXA-181_ (45.36 mol%) and *bla*_OXA-48_-like (44.49 mol%).

**Fig. 2.**
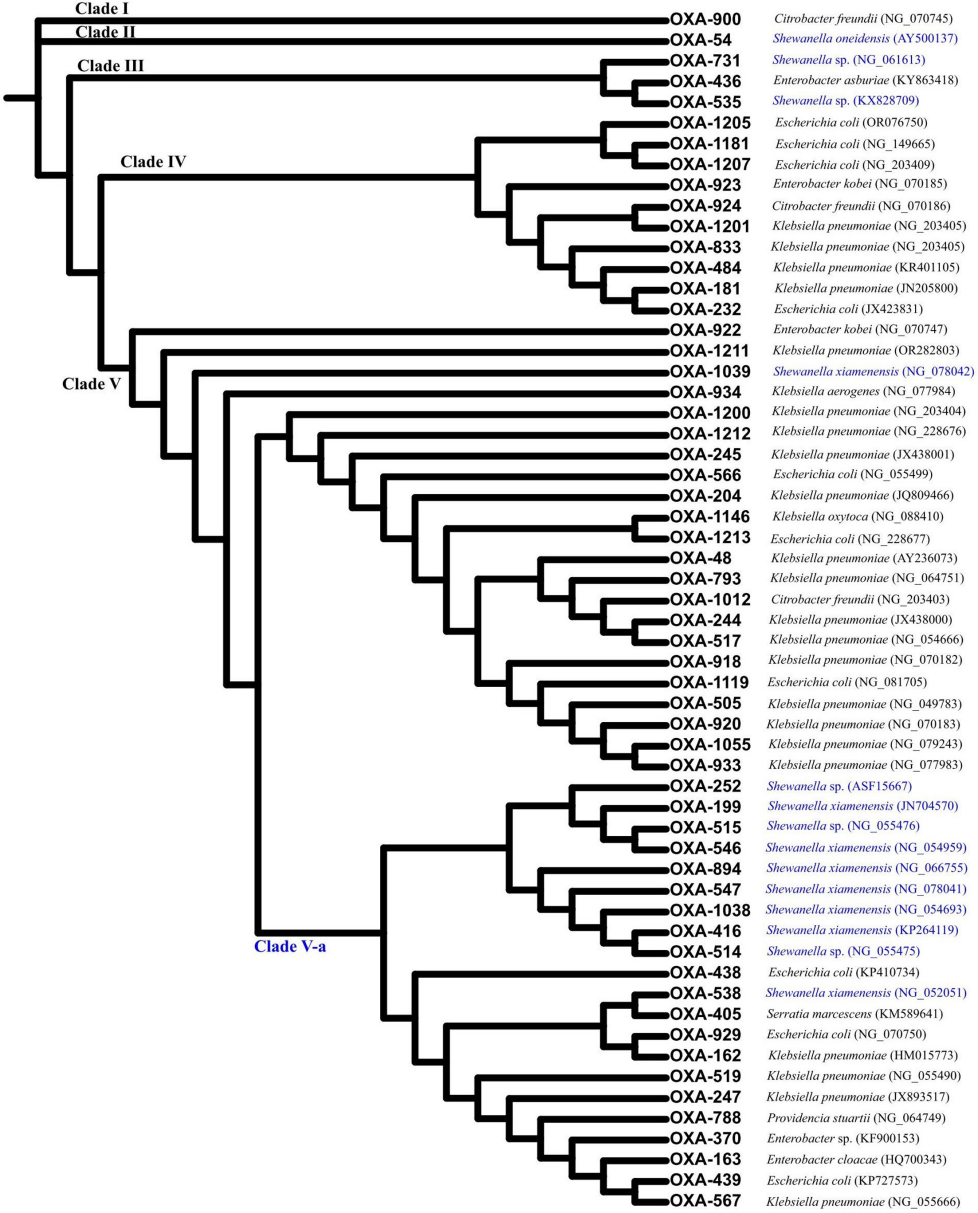
Phylogenetic tree of all OXA-48-like carbapenemases. Cladogram of the tree of *bla*_OXA-48_-like genes with clades annotated on the branches. A total of 58 OXA-48-like carbapenemases publicly available as of 1 December 2023 are included. The annotation alongside different variants indicates the species from which the corresponding OXA-48-like carbapenemase was reported and the accession number of the associated nucleotide acid sequence. The variants reported from *Shewanella* spp. are annotated with blue fonts. The amino acid sequence alignment of the 58 OXA-48-like carbapenemases is shown in Figure S1.

To test our hypothesis and determine the phylogenetic profile of OXA-48-like carbapenemase, multiple sequence alignment was performed on the amino acid sequences of a total of 58 OXA-48-like variants publicly available as of 1 December 2023. These variants shared 81.1–99.6% identity with OXA-48 *sensu stricto*. The reconstruction of an approximately maximum likelihood tree allowed us to identify five distinctive clades, the first two of which comprised only one variant, OXA-900 (Clade I) and OXA-54 (Clade II), which shared 81.1% and 92.5% identity with OXA-48, respectively (Fig. 2). Interestingly, OXA-900, proposed to be originated from *Shewanella* sp., was firstly reported from *Citrobacter freundii*, in which it was located on an antibiotic resistance island with mobile genetic elements on an IncC plasmid [38]. Clade V, which included OXA-48 *sensu stricto,* contained the highest number of OXA-48 variants (*n*=43). A subclade in Clade V, Clade V-a, comprised 21 OXA-48 variants reported from both *Shewanella* sp. and *Enterobacterales*, suggesting the close genetic relationship of such variants in these distantly related species. The remaining variants in Clade V were all reported from *Enterobacterales* except for OXA-1039, which was reported from *S. xiamenensis*, suggesting the further evolution of OXA-48-like carbapenemase after acquisition by *Enterobacterales*. Likewise, OXA-48 variants in Clade III (*n*=3) were from both *Shewanella* sp. and *Enterobacterales*, indicating the genus ancestor of *bla*_OXA-48_-like carbapenemase genes and its dissemination in *Enterobacterales*. Unlike these clades, OXA-48 variants in Clade IV (*n*=10) were all reported from *Enterobacterales*, including OXA-181 and OXA-232, the second and third most common global OXA-48-like derivatives [13] (Fig. 2).

### Species and carriage of carbapenemase genes of *Shewanella* spp. from different sources in Zhejiang province

A total of 38 *Shewanella* spp. were collected from different sources in Zhejiang province, including clinical settings (*n*=13), hospital sewage (*n*=14) and river water (*n*=11). Strains from the three sources were denoted with unique identifiers (IDs) beginning with C, F, and H, respectively. The 38 strains belonged to four different species, including *S. xiamenensis* (*n*=23), *S. algae* (*n*=12), * S. mangrovisoli* (*n*=2) and *Shewanella indica* (*n*=1). Interestingly, the *S. algae* and *S. indica* strains were all isolated from clinical settings. *S. xiamenensis* strains were isolated from both hospital sewage (*n*=13) and river water (*n*=10). Likewise, *S. mangrovisoli* was also isolated from both hospital sewage (*n*=1) and river water (*n*=1).

**Fig. 3.**
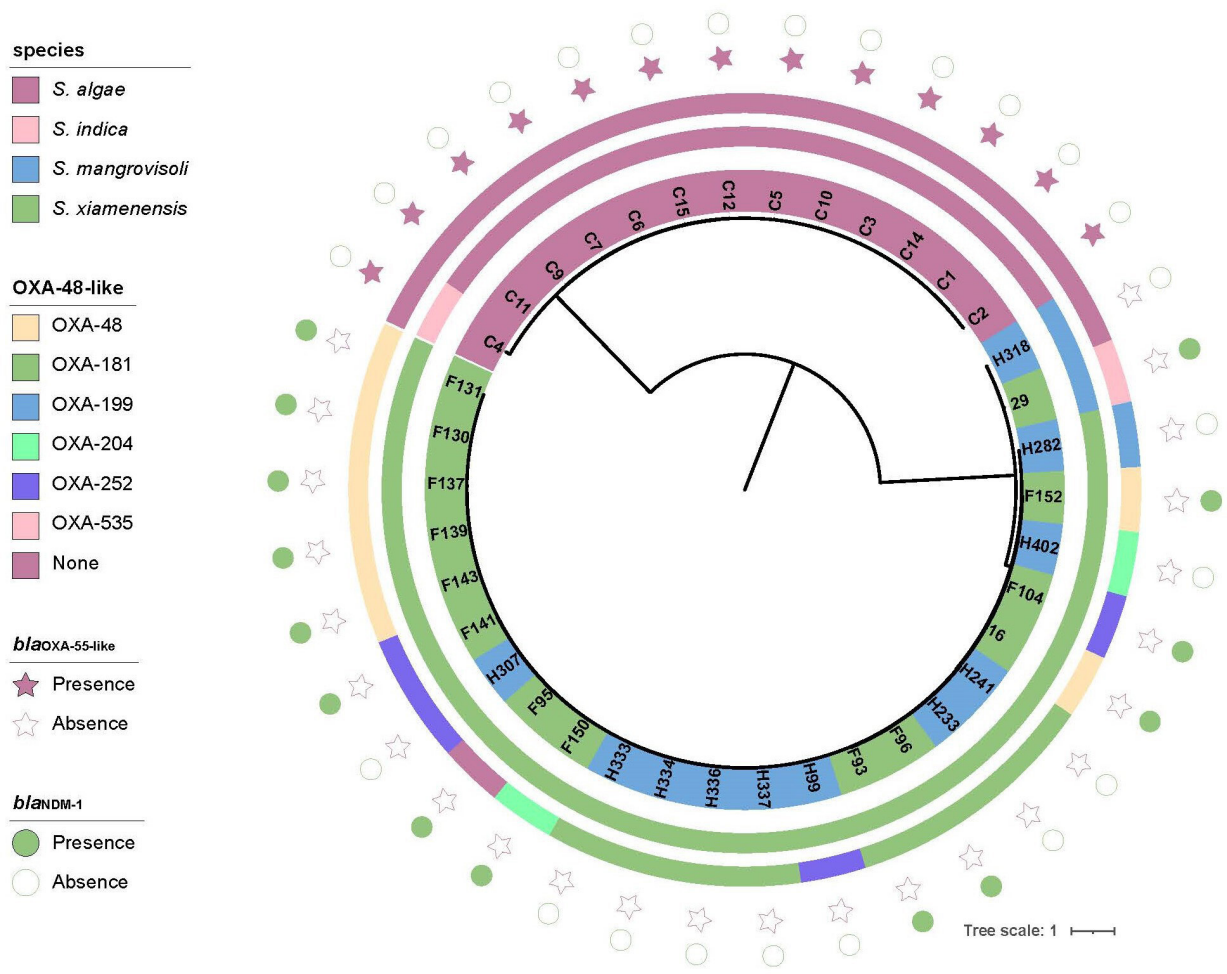
Phylogenetic tree of *Shewanella* spp*.* in this study. The background colour of the strains indicates the source of the corresponding strains which are from a clinical setting (pink), hospital sewage (green) and river (blue), respectively (strains from the three sources are denoted with IDs beginning with C, F and H, respectively). The innermost to outermost circles in this tree indicate the species, the production of OXA-48-like carbapenemase and the presence of the carbapenemase genes *bla*_OXA-55-like_ and *bla*_NDM-1_, respectively.

According to previous studies, the *bla*_OXA-55_ gene was intrinsic to *S. algae* [39]. Likewise, all the 13 clinical isolates carried the *bla*_OXA-55_-like carbapenemase genes. Yet, none of the clinical isolates carried the *bla*_OXA-48_-like gene. *S. mangrovisoli* strain H318 from river water did not carry any carbapenemase gene, and *S. mangrovisoli* strain 29 from hospital sewage carried both *bla*_OXA-535_-like and *bla*_NDM-1_ genes. All the *S. xiamenensis* from hospital sewage carried *bla*_NDM-1_, and none of the strains from river freshwater encoded the NDM carbapenemase. Like that of strains in the NCBI database, *S. xiamenensis* in our collection carried a wide diversity of *bla*_OXA-48-like_ carbapenemase genes. The dominant *bla*_OXA-48-like_ gene carried by the river water *S. xiamenensis* isolates was *bla*_OXA-181_ (*n*=6), followed by *bla*_OXA-252_-like (*n*=2), *bla*_OXA-199_-like (*n*=1) and *bla*_OXA-204_-like (*n*=1). The dominant *bla*_OXA-48-like_ gene carried by the *S. xiamenensis* isolates from hospital sewage was *bla*_OXA-48-like_ (*n*=7), followed by *bla*_OXA-181_ (*n*=2), *bla*_OXA-252_-like (*n*=2) and *bla*_OXA-204_-like (*n*=1). One * S. xiamenensis* from hospital sewage did not carry the *bla*_OXA-48-like_ gene. (Fig. 3, Table S4).

### Antimicrobial resistance profiles of *Shewanella* spp. in Zhejiang province

**Fig. 4.**
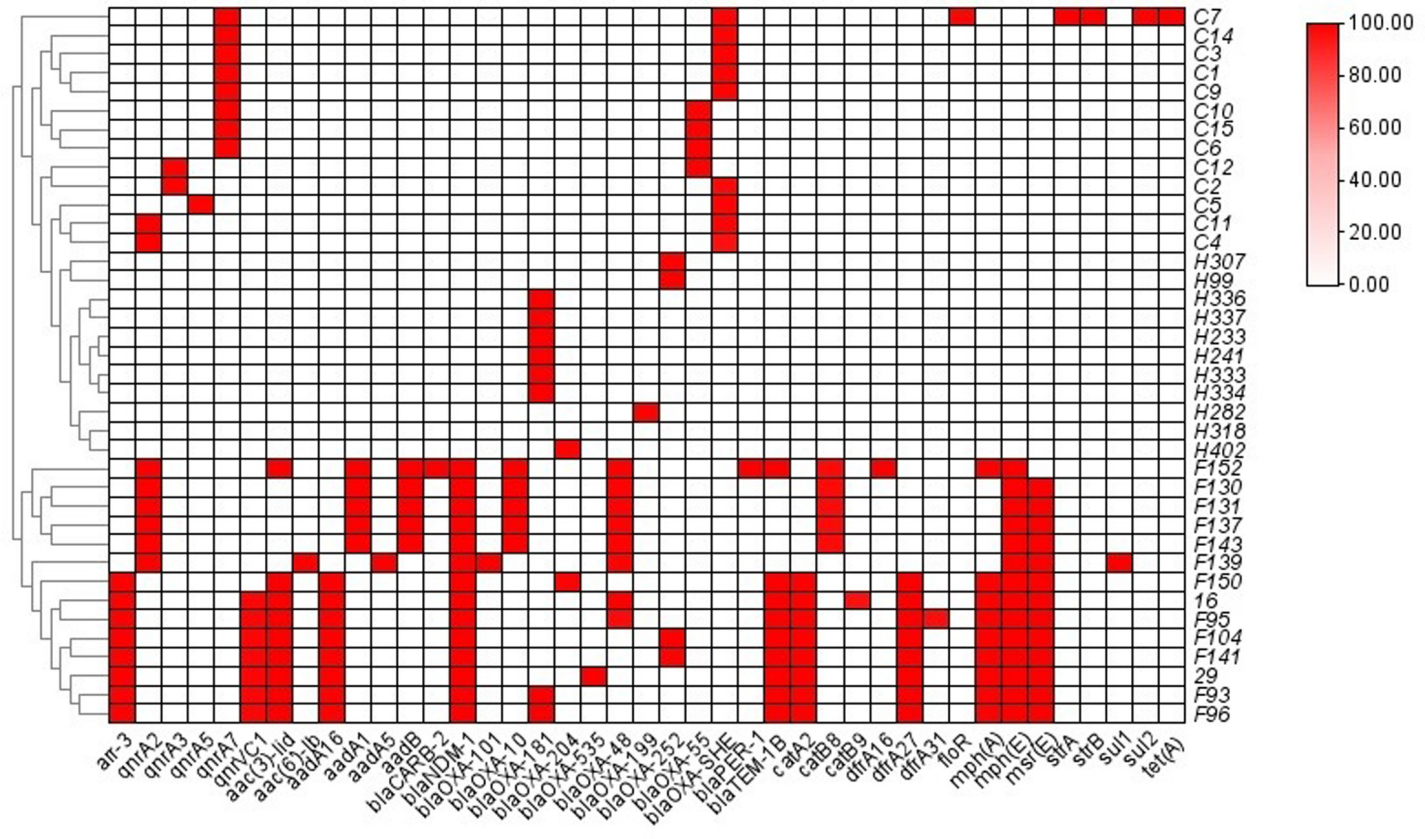
Heatmap of antimicrobial resistance genes carried by the *Shewanella* spp. in this study. Horizontal axes represent the antimicrobial resistance genes, and vertical axes represent the strain IDs. Red and white boxes represent the presence and absence of the corresponding items among sequenced isolates, respectively. The gradient identity bar indicates the percentage similarity of related genes. The similarity tree was calculated using agglomerative hierarchical clustering, with the degree of similarity between different clusters being calculated by the average linkage method and the degree of similarity of different isolates calculated with Spearman’s rank correlation coefficient.

A heatmap was plotted to depict the patterns of antimicrobial resistance genes carried by *Shewanella* strains in this study (Fig. 4). Our results suggested that the resistance genes carried by *Shewanella* exhibited a source-associated pattern. Strains from clinical settings and river waters carried fewer resistance genes compared with those from hospital sewage. Specifically, strains from the river carried no resistance genes apart from *bla*_OXA-48-like_. Apart from one strain, *S. algae* C7, carrying a cassette of resistance genes including *qnrA7*, *floR*, *strAB*, *sul2*, *tet*(A) and *bla*_OXA-55_-like, all other *Shewanella* strains from clinical settings carried only two resistance genes, i.e. a quinolone resistance gene *qnr* and a *bla*_OXA-55_-like gene. Additionally, SNP analysis revealed that the 13 * S*. *algae* strains had an average SNP count of 78,610.9 and a median of 55,888, indicating that they do not belong to the same clone. The number of resistance genes carried by isolates from hospital wastewater ranged from 9 to 14, conferring resistance to different classes of antibiotics including aminoglycosides, quinolones, macrolides, phenicols, rifampin, sulphonamides, trimethoprim and *β*-lactamases (Fig. 4, Table S4). In line with the resistance gene profiles carried by each strain, the *bla*_OXA-48_-negative strains were susceptible to commonly used antibiotics, and *bla*_OXA-48_-positive strains were resistant to antibiotics such as carbapenemase (Tables 1 and S5).

**Table 1.** Antimicrobial susceptibility profiles of *Shewanella* spp*.* in this study

Strainantibiotic	OXA-48-negative strain		OXA-48-positive strains	
	MIC_50_ (µg ml^−1^)	MIC_90_ (µg ml^−1^)	MIC_50_ (µg ml^−1^)	MIC_90_ (µg ml^−1^)
IPM	≤1	2	64	128
MEM	≤1	≤1	32	64
ETP	≤2	≤2	128	＞128
CMZ	≤2	4	≤2	16
CAZ	≤2	≤2	>128	>128
CTX	≤4	≤4	128	>128
TZP	≤8/4	≤8/4	≤8/4	≤8/4
SCF	≤8/4	≤8/4	≤8/4	≤8/4
CAV	≤0.5	≤0.5	≤0.5	≤0.5
FEP	≤4	≤4	16	64
PB	1	1	≤0.5	>8
TGC	≤0.25	≤0.25	≤0.25	≤0.25
CIP	≤1	2	≤1	4
AK	≤4	≤4	≤4	≤4
ATM	≤4	≤4	≤4	≤4

AK, amikacin; ATM, aztreonam; CAV, ceftazidime/avibactam; CAZ, ceftazidime; CIP, ciprofloxacin; CMZ, cefmetazole; CTX, cefotaxime; ETP, ertapenem; FEP, cefepime; IPM, imipenem; MEM, meropenem; PB, polymyxin B; SCF, cefoperazone/sulbactam; TGC, tigecycline; TZP, piperacillin/tazobactam.

### Genetic environment of *bla*_OXA-48_-like genes in Zhejiang province

**Fig. 5.**
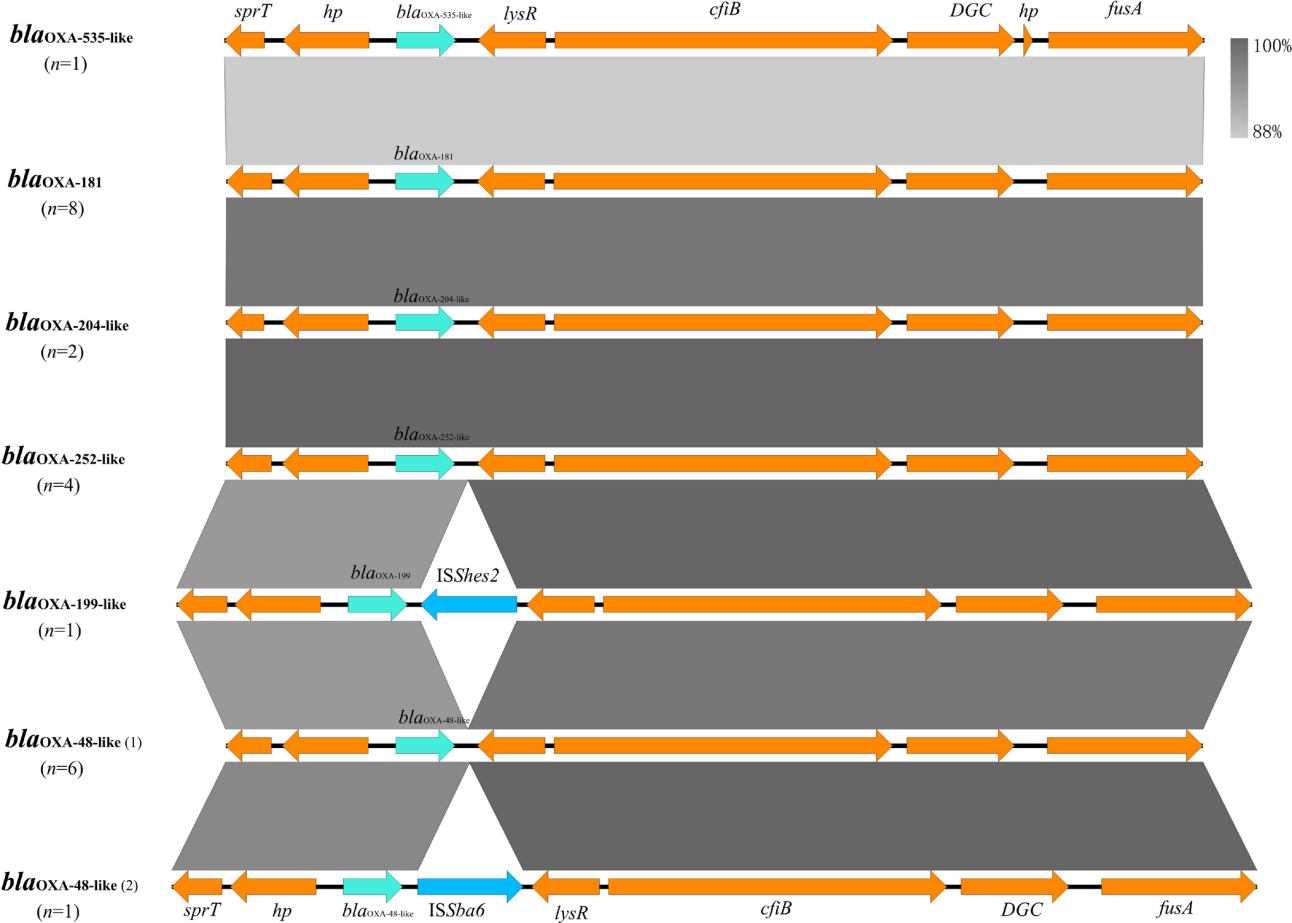
Genetic context of *bla*_OXA-48_-like genes in this study. Cyan, blue and orange arrows indicate the antibiotic resistance gene *bla*_OXA-48_-like, mobile genetic element and other ORFs, respectively. Shading areas indicate the region of genetic elements sharing sequence identities. A number of strains with the corresponding genetic arrangements are given in brackets. Subtypes of the *bla*_OXA-48_-like genes are labelled alongside the corresponding arrows.

Genetic analysis suggested that *bla*_OXA-48-like_ genes in all 23 *Shewanella* spp. were located on the chromosome. Despite variations in the sequences of *bla*_OXA-48-like_ genes, all *Shewanella* species examined harboured these genes at an identical chromosomal locus. The core genetic contexts of *bla*_OXA-48-like_ genes in *Shewanella* strains in this study were all *sprT-hp-bla*_OXA-48_-like-*lysR-cfiB-DGC-fusA*. Mobile elements were only observed in two strains inserted between the *bla*_OXA-48-like_ and *lysR* genes, one of which is *S. xiamenensis* H282 from river water carrying *bla*_OXA-199-like_-IS*Shes2-lysR*, and the other is *S. xiamenensis* F152 from hospital sewage carrying *bla*_OXA-48-like_-IS*Sba6-lysR* (Fig. 5). The presence of such genes could be associated with the mobilization of *bla*_OXA-48-like_ genes.

## Discussion

*Shewanella* sp. is an important environmental species playing pivotal ecological roles, with several members being pathogenic to human beings [40]. Strains in this genus were considered important carriers and reservoirs of carbapenemase genes, particularly the Ambler class D *β*-lactamase which they carry on chromosomes [41]. *Shewanella* sp. was reported to be the progenitor of *bla*_OXA-48_-like carbapenemase genes, which were among the major causes of carbapenem resistance in clinically significant pathogens within the *Enterobacterales* order [13,14]. The *Shewanella* genus contained a total of 130 species at the time of writing, and a total of 58 OXA-48 variants were reported to date. Although the progenitors of several OXA-48-like carbapenemases have been investigated, there has been no systematic study reporting the association between different *Shewanella* species and these carbapenemase variants. Besides, the molecular epidemiology of *Shewanella* in Zhejiang province remained largely unknown. To address these issues, we analysed the largest collection of *Shewanella* genomes to our knowledge and performed genetic and phenotypic analysis on *Shewanella* collected from Zhejiang province. However, this study has certain limitations. The initial identification of collected strains was performed using MALDI-TOF MS, yet the available MS libraries contain only a limited number of *Shewanella* species references. This constraint raises the possibility of undetected *Shewanella* species that might have been missed during the identification process.

To the best of our knowledge, this is the first study investigating the progenitor of different *bla*_OXA-48_-like variants with a focus on the *Shewanella* population. Results in this study showed that different *bla*_OXA-48_-like variants originated from different *Shewanella* species, and only some of the *Shewanella* species were associated with the *bla*_OXA-48_ genes. In particular, *S. xiamenensis* could be the progenitor of different *bla*_OXA-48_ variants [16,18]. However, it is worth noting that the predominance of *S. xiamenensis* and *S. algae* in our findings may be influenced by their relatively higher abundance in the sampled populations, while other *Shewanella* species might be underrepresented due to their rarity. *S. xiamenensis* is a zoonotic pathogen commonly found in the aquatic ecosystem and rarely isolated from clinical samples [42]. Reflecting this rarity, our study, which examined a limited number of strains, did not include any * S. xiamenensis* isolates from clinical samples, aligning with the general scarcity of such clinical reports [39,42]. Our phylogenetic analysis further demonstrated that *Shewanella* spp. are progenitors of the *bla*_OXA-48_-like genes [17]. Besides, OXA-48-like carbapenemases have undergone constant evolution in *Enterobacterales* and were highly diverse, with at least five clades detected [13]. In our study, which was conducted with a small sample size in Zhejiang, the distribution of different *Shewanella* species appeared to be niche-specific: *S. algae* (*n*=12) and *S. indica* (*n*=1) strains were exclusively isolated from clinical settings, while *S. xiamenensis* (*n*=23) and *S. mangrovisoli* (*n*=2) were found in both hospital sewage and river water. However, due to the limited number of isolates analysed, the conclusions drawn from this study should be interpreted with caution, acknowledging the potential limitations on the generalizability of our findings. None of the clinical *Shewanella* isolates carried the *bla*_OXA-48-like_ genes. Comparatively, all but one *Shewanella* isolate (*S. mangrovisoli*) from the water sample carried *bla*_OXA-48-like_. *bla*_OXA-48-like_ genes in *Shewanella* spp. from Zhejiang were located on the chromosome. Findings in this study supported the notion that OXA-48-like carbapenemase originated from different environmental *Shewanella* species and was captured by clinical species, particularly *Enterobacterales*. Findings of this research underscore the significant function of *Shewanella* species within ecosystems, especially as the predominant origin of the notorious carbapenemase gene, *bla*_OXA-48_. It is imperative to enforce preventive strategies to curb the spread of these organisms in both hospital environments and communal settings.

## Conclusions

In summary, our genomic analysis of all *Shewanella* strains in the database suggested that *bla*_OXA-48_-like was intrinsically carried by a few *Shewanella* species, and different *bla*_OXA-48_-like variants were associated with different *Shewanella* species. Particularly, the *bla*_OXA-48_-like genes carried by *S. xiamenensis* were highly diverse. Comparatively, none of the *S. algae* genomes carried *bla*_OXA-48_-like, although whether this result is influenced by species abundance requires further investigation. Our phylogenetic analysis of all OXA-48-like carbapenemases suggested they originated from different environmental *Shewanella* species and were captured by clinical species, particularly *Enterobacterales*. The distribution patterns of *Shewanella* species in Zhejiang province, with *S. algae* (*n*=12) and *S. indica* (*n*=1) isolated from clinical settings and *S. xiamenensis* (*n*=23) and *S. mangrovisoli* (*n*=2) from both hospital sewage and river water, should be interpreted in the context of our study’s limited sample size. *bla*_OXA-48-like_ genes in *Shewanella* spp. in this study were located on the chromosome. Our findings underscored the pivotal ecological function of *Shewanella* species, notably as the primary conduit for the widely recognized carbapenemase gene, *bla*_OXA-48_.

## Supplementary material

10.1099/mgen.0.001417 Uncited Fig. S1.

10.1099/mgen.0.001417 Uncited Supplementary Material 1.

## Abbreviations

ANI: average nucleotide identity
IDs: unique identifiers
ISs: insertion sequences
MICs: minimum inhibitory concentrations
NCBI: National Center for Biotechnology Information
