# Supplementary figures and images for: Genetic insights into Shewanella spp., progenitor of the blaOXA-48-like genes: a large-scale study
Source: Microb Genom. 2025 Jun 5;11(6):001417. doi: 10.1099/mgen.0.001417 (PMC12282331; doi:10.1099/mgen.0.001417)

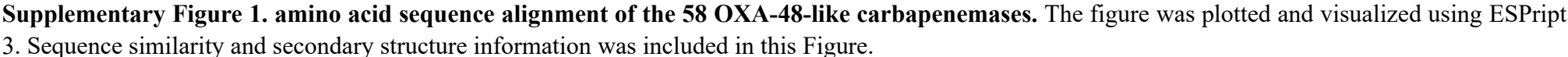

3. Sequence similarity and secondary structure information was included in this Figure.

Supplement: Uncited Fig. S1. [file mgen-11-01417-s001.pdf]
